# Health Impact of Street Sweeps from the Perspective of Healthcare Providers

**DOI:** 10.1007/s11606-022-07471-y

**Published:** 2022-03-16

**Authors:** Diane Qi, Kamran Abri, M. Rani Mukherjee, Amy Rosenwohl-Mack, Lina Khoeur, Lily Barnard, Kelly Ray Knight

**Affiliations:** 1 UCSF School of Medicine, San Francisco, CA USA; 2 UCSF School of Nursing, San Francisco, CA USA; 3 UCSF Department of Family Medicine, San Francisco, CA USA; 4 UCSF Department of Humanities and Social Sciences, San Francisco, CA USA

**Keywords:** Homelessness, Street sweeps, Street homelessness, Health disparity

## Abstract

**Background:**

Homeless street sweeps are frequent operations in many cities in the USA in which government agencies move unhoused people living in public outdoor areas. Little research exists on the health impact of street sweeps operations.

**Objective:**

This study was created at the request of community advocacy groups to investigate and document the health impacts of street sweeps from the perspective of healthcare providers.

**Design:**

This is a qualitative study using data gathered from open-ended questions.

**Participants:**

We recruited 39 healthcare providers who provided health and wellness services in San Francisco for people experiencing homelessness (PEH) between January 2018 and January 2020.

**Interventions:**

We administered a qualitative, open-ended questionnaire to healthcare providers using Qualtrics surveying their perspectives on the health impact of street sweeps.

**Approach:**

We conducted qualitative thematic analysis on questionnaire results.

**Key Results:**

Street sweeps may negatively impact health through two outcomes. The first outcome is material loss, including belongings and medical items. The second outcome is instability, including geographic displacement, community fragmentation, and loss to follow-up. These outcomes may contribute to less effective management of chronic health conditions, infectious diseases, and substance use disorders, and may increase physical injuries and worsen mental health. Providers also reported that sweeps may negatively impact the healthcare system by promoting increased usage of emergency departments and inpatient hospital care.

**Conclusions:**

Sweeps may have several negative consequences for the physical and mental health of the PEH community and for the healthcare system.

**Supplementary Information:**

The online version contains supplementary material available at 10.1007/s11606-022-07471-y.

## INTRODUCTION

On a single night in January 2020, there were 580,466 persons experiencing homelessness (PEH) in the USA according to the U.S. Department of Housing and Urban Development Point-in-Time (PIT) count. Among all PEH, 38.9% were unsheltered, and among unhoused individuals (those not accompanied by spouses or families), 51.2% were unsheltered.^1^ Homelessness is associated with worse health outcomes, and this disparity is amplified in PEH who are unsheltered.^2^ Housing First models, such as permanent supportive housing (PSH) that provides both housing and healthcare and social services (e.g., mental health care, substance use treatment, job training), both decrease the prevalence of PEH and improve health outcomes for PEH.^3–5^

However, despite recognition that accessible housing and supportive services help to decrease homelessness, there remains a large population of unsheltered PEH. US urban cities are increasingly using laws to regulate how PEH can access and use public spaces. Some claim these laws are a form of coercive care to improve well-being of PEH, while others have argued that these regulations are implemented to hide or remove PEH from urban communities^6–8^ due to concern of the negative impact of PEH on sanitation, safety of housed residents, property values, local businesses, and aesthetics.^6,7,9,10^

One method for regulating how PEH use public spaces is street sweeps, also referred to as “clean-ups” or “encampment resolutions.” Sweeps are actions in which government agencies, often in collaboration with the Police Department, move PEH out of the location where they are sleeping. These agencies justify these mandatory relocations by citing violations of city regulations that denote where PEH can sleep, with claims that these regulations are necessary for purposes such as public sanitation operations or to reduce interference with pedestrian traffic. ^11,12^ During these weekly to daily forced relocations, data from community organizations suggest PEH are rarely transitioned into alternative shelter, despite government claims.^13,14^ One community report in San Francisco found that 91% of PEH remained in a public space after being moved in a sweep.^13^ Moreover, items belonging to PEH are often confiscated and lost^13,15^. Due to their regularity, homeless sweeps have become a defining feature of the daily experiences of unsheltered PEH.^13,15,16^ Moreover, cities across the USA (including San Francisco, Los Angeles, Seattle, New York City, and Chicago) have increased street sweeps operations, now spending millions of dollars on these operations each year.^15,17–23^

While there is some research examining the psychosocial and civic impacts of homeless sweeps, there is no prior research examining the health impacts of homeless sweeps.^24^ In this qualitative study, we focus on San Francisco, where the January 2019 PIT count found a 14% increase in numbers of PEH since 2017, and where 64% of PEH were unsheltered.^25^ During the time of this study (Sep 2019–Jan 2020), San Francisco was operating routine street sweeps coordinated by multiple government agencies.^13,15,22,23,26^ We surveyed healthcare providers to elicit their observations about the health impacts of these sweeps.

## METHODS

### Sample

We recruited providers using purposive snowball sampling. Researchers first identified healthcare agencies known to serve a high volume of PEH. The questionnaire was distributed to these agencies, and providers could opt in to participate. Providers were eligible if they provided paid or volunteer health and wellness services in San Francisco for PEH between January 2018 and January 2020. We targeted healthcare providers in this study given their professional expertise in health. “Healthcare provider” was defined broadly to capture a wide range of perspectives. In total, we recruited 39 eligible providers spanning a range of professions and roles (see Tables 1 and 2). No compensation was provided to questionnaire respondents.

**Table 1:** Demographic breakdown of providers by profession. Total respondents *n*= 39, with 7 participants choosing to withhold demographic information

Profession	Number of providers (%) [*n*=32]
Medical doctor	13 (41%)
Case manager	5 (16%)
Executive/program director	4 (13%)
Community outreach worker	3 (9%)
Registered nurse	2 (6%)
Social worker	2 (6%)
Childcare provider	1 (3%)
Nurse practitioner	1 (3%)
Medical assistant	1 (3%)

**Table 2:** Demographic breakdown of participants by field of work. Percentages add up to > 100% because some providers listed multiple fields of work

Field of work	Number of providers (%) [*n*=32]
Outpatient Medicine	10 (31%)
Community Health Centers	9 (28%)
Outreach Organizations	7 (22%)
Inpatient Medicine	5 (16%)
Street Medicine	4 (13%)
Psychiatry	2 (6%)
Emergency Medicine	1 (3%)

### Questionnaire

A qualitative questionnaire method was chosen to capture a breadth of perspectives from a larger sample of healthcare providers working in different settings. The questionnaire was designed with iterative feedback from research experts in the field of housing and homelessness and from members of community organizations working with PEH. The questionnaire was created via Qualtrics and included 9 open-ended questions that assessed how street sweeps may have impacted the medical care and health of PEH, from providers’ perspectives, and addressed the following topics: descriptions of belongings lost in sweeps and the impacts of those losses on clients/patients; impacts of sweeps on client/patient well-being and medical care; impacts of sweeps on the healthcare systems; what providers would like to convey to city officials about the impact of sweeps; and providers’ suggestions for policy alternatives to sweeps.

 Providers were able to opt out of responding to any question. The full questionnaire can be found in Appendix.

### Analysis

Thematic analysis of questionnaire responses was performed using Dedoose software by four members of the study team. First, responses to each question in the questionnaire were independently coded by research team members using inductive coding. After independent coding, a shared codebook was created and refined through a discussion and consensus process to reflect the emerging themes from the responses. All four coding team members then reviewed the full dataset and codebook together to agree on a final coded version of the questionnaire data. Finally, the codes were collated into the set of themes reported below, through an iterative process of team discussion and memoing. ^27,28^

## RESULTS

**Figure 1:**
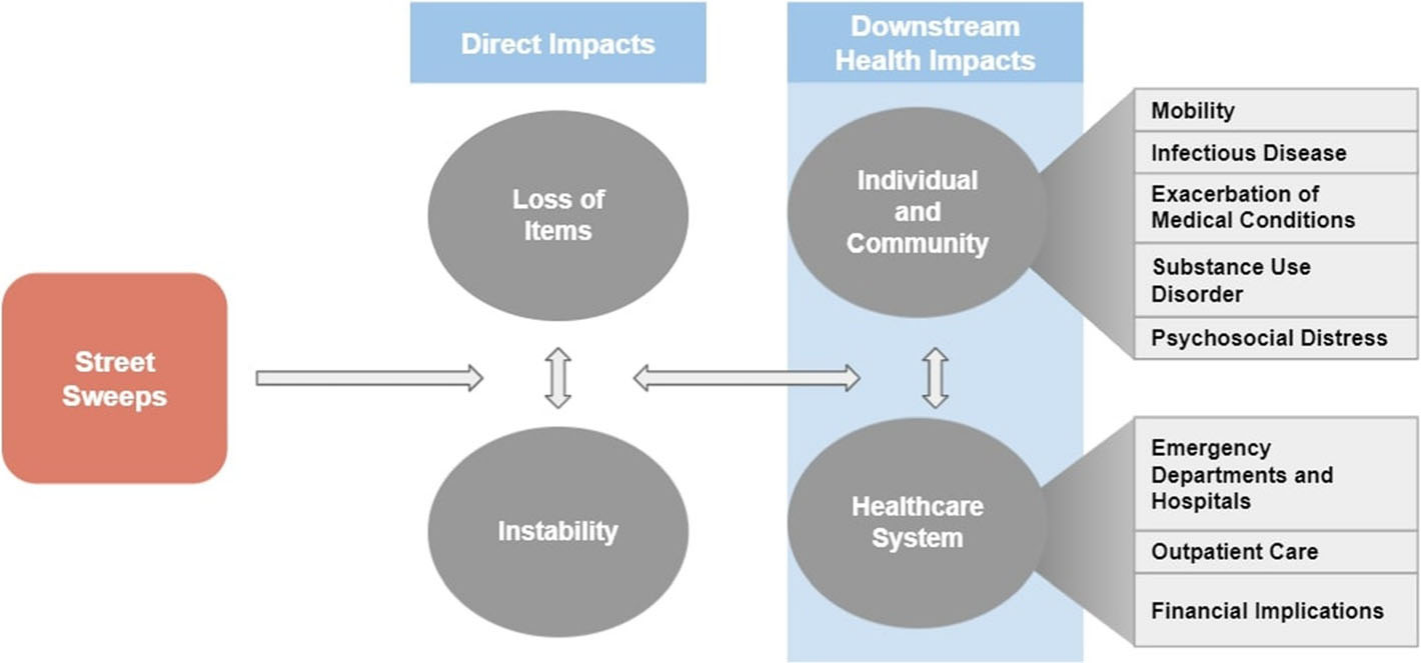
Visual representation of the relationships between street sweeps, direct health impacts, and downstream health impacts

Providers noted two direct outcomes resulting from street sweeps: material losses and instability. These two outcomes may have downstream impacts on individual health and health systems (Fig. 1).

### Direct Impacts

#### Material Losses

All providers but one discussed material losses due to street sweeps: “It’s like you come home from work and the house you’ve lived in for years has burned down and you have nothing now. All your memories and everything that is near and dear to you is gone” (Outreach Worker).

##### Medical Items

The most frequently specified medications were medication-assisted treatment for opioid use disorder, naloxone, hepatitis C, and HIV medications. Providers also noted loss of sterile injection equipment and medical devices, including durable medical equipment such as walkers, wheelchairs, canes, and crutches. Providers described that lost medical items are difficult to replace:

It is extremely difficult to get medications replaced!! Insurance companies… do not want to replace lost or stolen medications and this often takes many phone calls, paperwork, extensive advocacy to get people their medications--often resulting in the patient not being on medication for weeks to even months (Medical Doctor).

##### Items for Shelter and Daily Living

Providers discussed loss of sentimental items, survival gear, food, phones, money, and hygiene items. Sentimental items included mail, photographs, children’s items, family heirlooms, and loved one’s ashes. Survival gear included tents, backpacks, blankets, and tarps. Several providers described the health impacts of losing these items: “Loss of tents (shelter) and clothes and sleeping bags can make people who are already ill much more sick, and risk them needing hospitalization or, failing that, dying” (Program Manager, Community Health Organization).

#### Instability

Providers discussed three types of instability that may be caused by street sweeps.

##### Geographic Instability

Providers described geographic instability due to disrupted living location: “A person cannot rely on setting up a place they can rest; it causes greater discomfort, insecurity, and instability. These things snowball” (Registered Nurse).

##### Community Instability

Providers also discussed the community instability caused by “dispersing encampments.” One physician discussed these protective factors of community, noting that encampments enable people to better perform activities of daily living and to create networks of trust to support their health and livelihoods:

I am most concerned about encampments that have a sense of community [that] provides security; they can leave their stuff and work or go to important appointments; there are people they can count on to help them, sometimes rescue them [from opioid overdose] with Narcan^TM^ [naloxone]; they share food. (Medical Doctor)

##### Follow-up Instability

Finally, providers noted that sweeps cause the loss of a stable location for PEH to receive follow-up medical care: “The sweeps make providing healthcare to individuals who live outside very difficult…I am unable to find people after they have been moved” (Registered Nurse).

### Downstream Health Impacts

The direct impact of sweeps may lead to several downstream health impacts, both on PEH and the healthcare system.

#### Impact on PEH Individuals and Community

##### Mobility

Providers found that sweeps may decrease mobility through loss of mobility equipment such as walkers, which can prevent access to services. For example, one provider noted that “Loss of mobility devices also may keep an individual from making it to see a provider, missing meals or food pantry opportunities, and other basic needs may go unmet” (Outreach Worker). In addition, impaired mobility increases risk of injuries (e.g., falls) and increases vulnerability to violence from others: “Getting a walker/wheelchair is very hard. People become immobilized, and are at risk for violence from others, injury, as well as exposure to the elements” (Role and setting not disclosed).

##### Increased Spread of Infectious Disease

Sweeps may interfere with the management of infectious diseases such as HIV, HCV, sexually transmitted infections, and skin and soft tissue infections. Providers noted that effective management of infectious disease requires consistent use of medications for sufficient duration. In addition, providers noted that inconsistent or incomplete medication use builds medication resistance of infectious organisms within communities:

Loss of retroviral medications along with increased barriers to engagement with medical providers [leads] to higher rates of transmission of HIV and Hepatitis C, creates medication resistance (starting/stopping medications causes this), [and results in] higher rates of other infections due to compromised immune systems. (Outreach Worker)

##### Exacerbation of Chronic Health Conditions

Providers noted that stress, loss of medical and other items, and disruption to health and social services from sweeps may negatively impact management of multiple chronic health conditions (listed in Table 3) leading to increased severity and frequency of acute exacerbations or complications, which may require hospitalization.

**Table 3:** Chronic health conditions exacerbated and/or complicated by street sweeps, according to participants

**Chronic health conditions**	
Cardiac and pulmonary	Hypertension, congestive heart failure, asthma
Neurologic	Chronic pain, seizure disorder
Psychiatric and behavioral	Major depressive disorder, generalized anxiety disorder, bipolar disorder, schizophrenia, substance use disorder
Endocrine	Diabetes

##### Substance Use

Providers described how loss of medication-assisted treatment, such as buprenorphine, can lead to withdrawal and increased substance use. Loss of safer use supplies, such as sterile syringes, naloxone, and fentanyl test strips, can increase risks of overdose and exposure to infectious disease:

People get really sick [from opioid withdrawal] without their meds, and it often drives them to go purchase illegal drugs in order to self-medicate. Losing injecting equipment makes people share their needles and other items and increases their risk for exposure to things like HIV and HCV. (Executive Director, Community Health Organization)

Moreover, the emotional suffering induced by frequent sweeps can increase substance use as a coping mechanism:

[Sweeps are] devastating to the patient/client's physical wellbeing and triggers [substance use] to cope. We often see increases in medical emergencies and overdosing after a sweep. (Outreach Worker)

##### Psychosocial stress

Providers found that sweeps may worsen mental wellbeing by causing depressive symptoms, anxiety, fear, stress, a sense of dehumanization, loss of trust and security, hopelessness, and loss of motivation:

[U]nhoused community members talk of their increased feelings of stress and anxiety, feelings of hopelessness, sadness (especially in connection to lost and irreplaceable items important to the individual) and decreased motivation. (Outreach Worker)

Providers also described sweeps as repetitive traumatic experiences for PEH:

Sweeps keep patients/clients in crisis mode and add to their history of traumatic experiences. Sweeps are chronic traumatic events in their lives. Patients/clients worry about potential sweeps, [are] triggered by actual sweeps of invasion of their personal space and struggle with the aftermath of confiscated belongings. All these experiences reinforce lack of trust in community and San Francisco institutions, [increase] depression and substance use… (Outreach Worker)

This creates a high-stress environment that may encourage survival-based, crisis-driven decision making: “Loss of tents and sleeping bags and clothes leads to [...] desperate, needs based choices that may not be what people would usually do to survive” (Program Manager, Community Health Organization). The mental suffering of sweeps can also decrease hope for the future and motivation for self-care, further impairing one’s capacity for managing physical and mental health conditions:

A person can end up feeling like it's impossible to even try… many of my patients expect the world to be designed to make them fail. [T]he sweeps, which force people to give up a small piece of safety and stability they have created, reinforce this view. This can lead people to feeling like there is no point in trying to obtain stability. (Registered Nurse)

#### Impact on Healthcare Systems

##### EDs and Hospitals

PEH may utilize EDs and inpatient services at higher rates following street sweeps due to increased incidence of acute medical exacerbations and to replace medical items lost in a street sweep.

Medications have been taken in sweeps; patients with chronic medical conditions need to take medications regularly and, if their medications have been taken, their health conditions get worse and result in their having to be seen in emergency departments and/or be hospitalized. This is costly and detrimental to their health. (Role and setting not disclosed)

##### Outpatient Care

Providers noted that street sweeps may decrease outpatient care utilization due to decreased mobility, exacerbations of medical conditions, increased substance use, and instability. Moreover, loss of documents such as appointment reminder cards and lost phones can be barriers to outpatient follow-up. Finally, fear of unattended items being taken during sweeps can make PEH reluctant to attend medical appointments:

Many of the patients in the clinic are in constant fear of their belongings and/or medications being stolen or lost in sweeps if they are away from their designated location for too long. This frequently results in their reluctance to remain for medical visits out of fear that their belongings will be missing when they return. (Medical Doctor)

##### Financial Implications

Providers noted that increased ED and inpatient hospital utilization is costly to the medical system. In addition, there may be increased healthcare cost to replace medical items lost in sweeps, both due to the cost of the items and the time it takes to replace items:

Patients lose HIV medications, Hep C medications due to street sweeps. This... is more costly to public insurance programs in the long run for having to pay for medications again when it was a preventable issue. (Clinical Social Worker)

### Messages to the City

Providers had several suggestions for how the city should address homelessness and create alternatives to sweeps.

#### Concerns and Recommendations

Almost all providers highlighted the negative aspects of sweeps. The most cited concerns characterized sweeps as inhumane and in violation of human rights. Of note, one provider shared that the coercive nature of sweeps is not intrinsically negative. Rather coercion can have a role at times in the continuum of care to “break a cycle of severe social instability” (P44, Medical Doctor). However, other providers highlighted that sweeps are not an effective component of the continuum of care as sweeps create rapid cycles of displacement and reestablishment of encampments rather than lasting change:

Just driving people from place to place and confiscating their belongings does nothing whatsoever to solve the problem [of homelessness]. (Director, Legal Aid Service Provider)

Many providers identified that “NIMBYism” (“Not In My Back Yard,” describes opposition by residents to construction projects or service provision designed to serve PEH in their neighborhood) drives sweeps, and that the city’s focus on short-term solutions like sweeps prevents investment in long-term solutions like housing.

Some providers suggested that sweeps be improved by better safeguarding items belonging to PEH. It was also noted that these procedures are supposed to be happening already: “They [the city] say they do this but they just say everything is trash and that's how they get around their own rules” (Outreach Worker).

In addition, instead of punitive responses to homelessness, providers recommended a trauma-informed approach incorporating harm reduction principles:

There needs to be a harm-reduction approach so that if people opt to live outside we respect that decision and help them to stay safe in doing so.” (Program Director, Community Social Service Non-Profit Organization)

#### Alternatives to Sweeps

##### More Housing/Shelter

Providers suggested housing as a key alternative to sweeps, including permanent supportive housing and temporary shelters such as navigation centers and overnight shelters. In addition, providers suggested offering sanctioned encampments with services until there is sufficient housing.

[The City should] create areas…where camping is allowed and services…[are] provided, such as clean water, waste disposal, restrooms and electricity. This done under the harm reduction philosophies would decrease the harms on our unhoused community members, increase health and wellness, improve outcomes with treatments provided and reduce overall costs. (Case Manager)

##### Improved/Expanded Resources

Providers suggested opening storage facilities for PEH, as well as increasing daytime drop-in centers, mental health and substance use treatment services, case management services, and reduction of barriers to accessing services (e.g., providing medical services at encampments). Others recommended expanded service provider training, including de-escalation skills and mental health and substance use awareness.

## DISCUSSION

In this study, healthcare providers working with PEH observed that sweeps may negatively impact the health of PEH through the loss of health-related items. This phenomenon has been well documented nationally by media and advocacy groups, but has not yet been described in medical literature^15,17,20,21,23,26,29^.

Material losses from sweeps may cause less effective management of chronic health conditions, infectious diseases, and substance use disorders, and loss of mobility devices may lead to increased risk of injuries. Of note, inadequate management of infectious diseases has population-level consequences, as poor management of infectious disease in one person increases the risk of widespread, medication-resistant infectious disease. This is compounded with the possible link between sweeps and chaotic drug use and overdose, as medications for opioid use disorder and safer use supplies including clean needles are lost during sweeps.

Another way that sweeps may have negative health impact is through the ecosystem of instability created by sweeps. This instability includes geographic displacement, community fragmentation, and loss to follow-up. Our finding that instability due to street sweeps may have downstream negative health consequences for PEH builds on literature on residential transience, defined as frequent displacement between unstable and temporary living situations. Studies have found that increased residential transience among low-income sheltered individuals is associated with increases in depression, suicidal ideation, and HIV risk behavior.^30–32^ More research is needed to examine the impact of residential instability among PEH who are unsheltered.

Moreover, providers reported that sweeps can worsen mental health. The confrontation, dehumanization, and instability induced by sweeps are traumatic experiences for a community that already has a high trauma load, including a high prevalence of adverse childhood experiences (ACEs) and PTSD.^33,34^ The trauma of sweeps can lead to increased chaotic substance use as a coping mechanism. Further, sweeps can disrupt trust between PEH and city organizations, leading to an antagonistic and unpredictable living environment that necessitates survival- and crisis-driven decision making. This environment can further distance PEH from the ability to envision and work toward long-term wellbeing and stability.

These findings support the theory of sweeps as a form of “pervasive penalty” where PEH are forced into “consistent punitive interactions with state officials” that harm PEH and perpetuate the social and economic conditions that lead to homelessness.^35^ This cycle can justify the need for more punitive measures without ameliorating the conditions that create homelessness, nor addressing its negative health impacts. In the state’s response to homelessness, there is continued deployment of sweeps while other actors, including government-associated agencies, simultaneously attempt to provide social and health services. These contradictory actions may contribute to what Lopez (2020) refers to as the “necropolitics of care” by which marginalized people are subjected “simultaneously to both compassion and brutality, or to brutality as a gatekeeper or even penance for access to health and social interventions.”^36^

Lastly, providers note that the negative impact of sweeps extends to the larger healthcare system. Providers suggest that sweeps can disrupt continuity of care at all levels in a costly and ineffective way: as access to low-acuity care is reduced by sweeps and their sequelae, utilization of expensive emergency and high-acuity care increases. Thus, sweeps may be an additional factor contributing to the well-documented high utilization of high-acuity services by PEH.^37^

Our study has several limitations. First, our study does not include PEH’s perspectives on the health impact of sweeps, nor was input from PEH obtained in designing this study due to lack of funds for appropriate compensation. While community organizations provided input on study design, lack of PEH perspective may have led to gaps in study design and study findings. Moreover, we did not include individuals involved in sweeps operations (e.g., law enforcement, city workers) in this study, thus their perspectives on the health impacts of sweeps on people experiencing homelessness are not reflected here. Furthermore, the study responses may reflect selection bias because providers opted in to completing the questionnaire. Lastly, this study took place in a single city with health care providers from care settings that serve large volumes of PEH which may limit generalizability.

Given the lack of medical literature on sweeps, we believe our study can help guide future investigation. Further research is needed to quantitatively explore any causal associations between sweeps and health consequences and to examine their generalizability in multiple locations. In addition, future studies may benefit from enrolling multiple additional stakeholders involved in sweep operations, including PEH, in order to assess similarities and differences in perspective on health impacts. Lastly, further research exploring specific areas of health impact may be valuable. For instance, there is need for further investigation of mental health impact which could utilize more targeted sampling of people with specific expertise in mental healthcare.

In conclusion, sweeps may have significant negative consequences for the physical and mental health of PEH and for the health care system. In the context of sweeps, how cities regulate public spaces may have direct public health consequences. Clinicians should consider homeless sweep policies as an additional social determinant of health for the PEH community, and advocate for alternatives to sweeps including increased supply of housing and shelter as well as medical and social services for PEH.

## Supplementary Information

ESM 1 (DOCX 20 kb)
