# Supplementary material for: Health Impact of Street Sweeps from the Perspective of Healthcare Providers
Source: J Gen Intern Med. 2022 Mar 16;37(14):3707–14. doi: 10.1007/s11606-022-07471-y (PMC9585118; doi:10.1007/s11606-022-07471-y)
Supplement: Supplementary file 1 — (DOCX 20 kb) [file 11606_2022_7471_MOESM1_ESM.docx]

Appendix A

**Health Impacts of Street Sweeps: Provider Perspectives Survey**
Survey Introduction:

In January 2018, San Francisco opened the Healthy Streets Operations Center (HSOC) which mainly coordinates the Department of Public Works (DPW) and the San Francisco Police Department (SFPD) in responding to "unsheltered homelessness and unhealthy street behaviors." Since then, HSOC has carried out street sweeps with increased frequency.

Street sweeps describe the event in which city officials move individuals and/or encampments out of public spaces and confiscate their belongings. The sweeps target residents who are currently unhoused, and are driven in part by service requests placed by San Francisco residents who wish for individuals or belongings to be removed. These belongings include tents, clothing, blankets, phones, medications (e.g. insulin, HIV meds, bupe), and medical equipment (e.g. walkers).

We recognize that there are differences in opinions about street sweeps. If you would like to learn more, links will be provided at the end of the survey.

**Through this survey, we would like to better understand the physical and mental health impacts of these sweeps from the perspective of medical providers. We thank you in advance for your time and participation.**

In this survey, we will collect responses anonymously. If you are willing to do an in-person interview (which will be reported confidentially), we will provide an opportunity to collect contact information at the end of the survey, on an unlinked, separate, survey form. The full information sheet for the entire study is included [here.](https://drive.google.com/file/d/1ekCB3M0X7iWjOQ9N-WNqxYG47EjTocn0/view?usp=sharing)

Survey:

1. What is your health role? (e.g. LCSW, curanderx, acupuncturist, MA, psychiatrist, NP, PharmD, PA, DO, etc.)

2. What is your professional setting? (e.g. acute care, ED, outpatient, outreach program, street medicine, private practice, etc.)

3. It is often helpful to link specific respondents to specific anecdotes. Would you be willing to have your title (e.g. physician, LCSW) and professional setting linked to your statements?

4. Have you provided patient/client care in San Francisco since January 2018?

5. Have you provided care for patients/clients who have been unhoused?

6. Have your patients/clients been affected by street sweeps? (e.g. individual being asked to move, personal items being confiscated, or larger encampments being cleared)

7. Have your patients/clients had their belongings taken during sweeps?

8. Please describe any belongings your patients/clients have lost to sweeps.

**Belongings taken during sweeps may include clean needles/injecting equipment, Narcan, and medication-assisted treatment such as buprenorphine/suboxone.**

9. Have your clients/patients been affected by loss of these items?

10. In your experience, what are the consequences of the loss of these items?

11. In your experience, how do sweeps and/or confiscation of resources impact the**medical care** of patients/clients?
(i.e. management of chronic conditions, healthcare delivery, communication, medication adherence)

12. In your experience, how do sweeps and/or confiscation of resources impact the **wellbeing** of patients/clients?
(i.e. mental health, physical health, safety, access to services, food security, etc.)

**There have been reports of individuals having medications taken during sweeps including insulin and anti-retroviral therapy. In addition, people have lost medical equipment such as walkers.**

13. How do you feel this may impact patient/client health?

14. How do you feel this may impact medical care and the health system?

**We are interested in hearing your perspective about potential solutions and alternatives.**

From your perspective, as a health and care provider, what would you like to convey to city officials (e.g. the mayor's office, DPW, SFPD) about the impacts of street sweeps?

15. Are there any solutions you think might work as an alternative to sweeps? For example:

- Suggestions for alternative allocation of funds that might address homelessness for unsheltered people who are experiencing sweeps
- Policy recommendations that might improve the health and well-being of people experiencing homelessness?

16. Please describe alternatives, if possible.

17. We would like to be as comprehensive as possible in our data collection. Please provide the names of any San Francisco colleagues who you feel might be interested in hearing about this research.

18. Any questions or concerns?
